# Late delayed radiation-induced cerebral Arteriopathy by high-resolution magnetic resonance imaging: a case report

**DOI:** 10.1186/s12883-019-1453-9

**Published:** 2019-10-02

**Authors:** Huan Chen, Xiuhua Li, Xiaoyu Zhang, Wenjuan Xu, Fei Mao, Mengxin Bao, Meijia Zhu

**Affiliations:** 1 Department of Neurology, Shandong University, First People’s Hospital of Jinan, Jinan, 250013 China; 2 Department of Neurology, Affiliated Qianfoshan Hospital of Shandong University, Jinan, 250014 China; 3 Department of Neurology, Liaocheng People’s Hospital, Liaocheng, 252000 China

**Keywords:** Radiotherapy, Arteriopathy, Vasculitis, High-resolution magnetic resonance imaging

## Abstract

**Background:**

Radiation therapy can cause cerebral arteriopahty, resulting in ischemic stroke. We document late-delayed cerebral arteriopathy by high-resolution magnetic resonance imaging (HR-MRI) in a middle aged man who had cranial irradiation 19 years earlier.

**Case presentation:**

A 45-year-old man was diagnosed with frontal lobe glioma 19 years ago and was treated with radiation after surgical resection. He was admitted to our hospital with an acute cerebral infarction in November 8, 2017. Traditional MRI examination and HR-MRI (sagittal, reconstruction of coronal and axial) were performed at admission. He was treated with prednisone (30 mg/day) and clinical symptoms disappeared after 3 months by telephone follow-up. Our patient complained of dizziness and blurred vision and traditional MRI examination indicated acute ischemic stroke in temporal lobe and occipital lobe and microbleeds. In order to define the exact mechanism of stroke, blood tests, auto-immune screening and thrombophilia were performed and results were normal. Electrocardiography and echocardiography were negative and cardiogenic cerebral embolism was excluded. In cerebrospinal fluid (CSF) examination, level of albumin and IgG were elevated. HR-MRI showed vessel wall thickening in T1-weighted imaging, narrow lumen in proton density imaging and vessel wall concentric enhancement in contrast-enhanced T1- weighted imaging. Combined with radiotherapy history, the patient was diagnosed with radioactive vasculitis.

**Conclusion:**

Radiation-induced cerebrovascular damages could be a lasting progress, which we cannot ignore. HR-MRI can provide sensitive and accurate diagnostic assessment of radiation-induced arteritis and may be a useful tool for the screening of causes of cryptogenic stroke.

## Background

Radiotherapy is an effective treatment for patients with intracranial tumors. Radiation-induced damage to brain has received increasing attention, including acute, early delayed and late delayed brain injury. Delayed radiation-induced damage in extra-cranial arteries has been well recognized [1, 2]. However, reports of radiation-induced intracranial vascular abnormalities were rare because of limited assessments. High-resolution magnetic resonance imaging (HR-MRI) is a vessel wall imaging technique which can directly evaluate intracranial vascular damage [3–6]. Here, we report a patient with radiation-induced cerebral arteriopathy with the use of HR-MRI and which is still progressing 19 years after radiotherapy.

## Case presentation

**Fig. 1:**
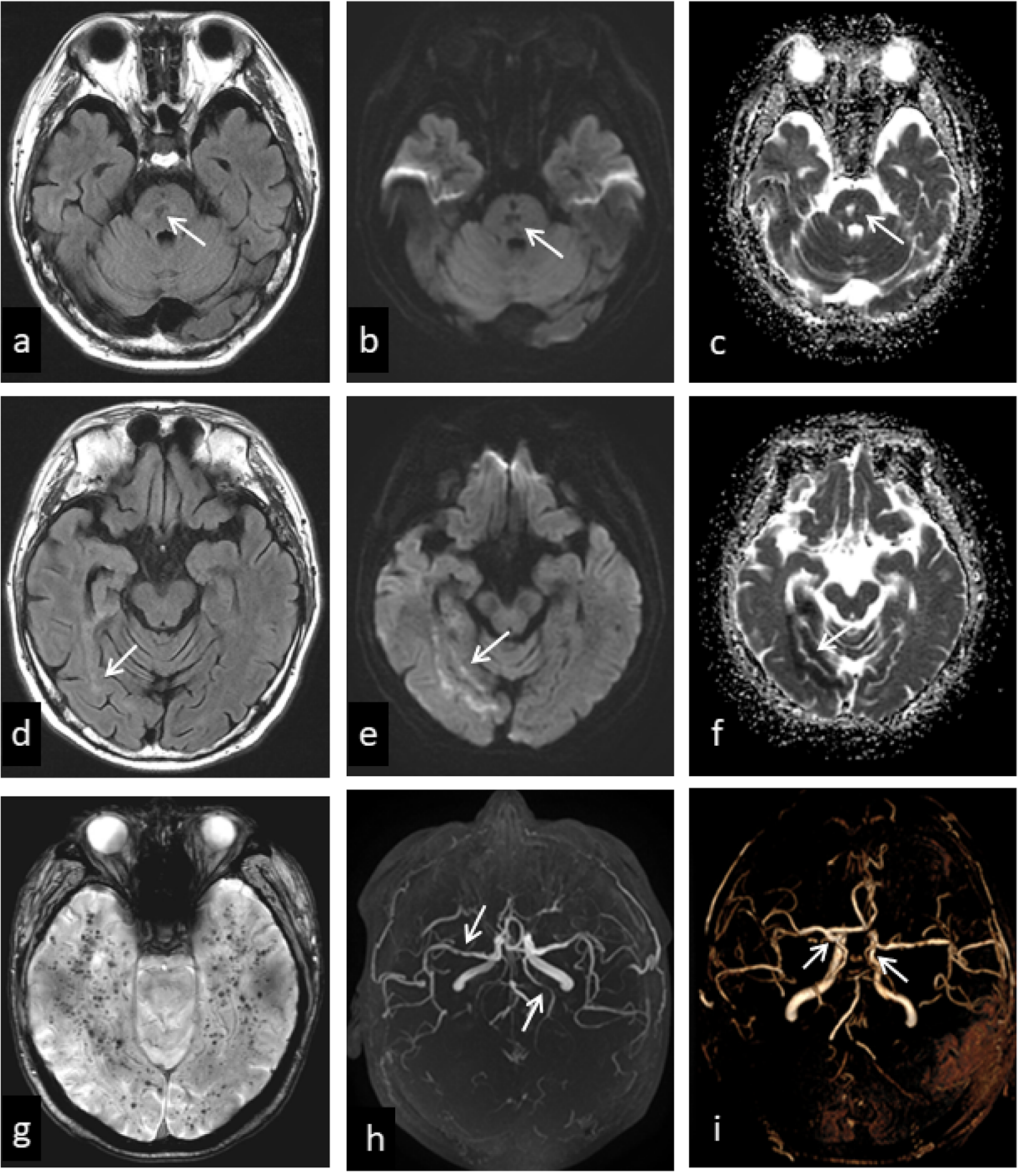
Brain magnetic resonance imaging (MRI). **a**-**c**: Infarction in brainstem with hypointense signal in fluid attenuated inversion recovery (FLAIR), hypointense in diffusion weighted imaging (DWI) and hyperintense signal in apparent diffusion coefficient (ADC). **d**-**f**: Acute ischemic stroke in temporal lobe and occipital lobe with relatively low signals in FLAIR, hyperintense signal in DWI and hypointense signal in ADC. **g**-**i**: CMBs in the susceptibility weighted imaging (SWI) sequence. Multiple localized stenosis of the right middle cerebral artery and the left posterior cerebral artery in MRA. The bilateral posterior cerebral artery originated from the bilateral internal carotid artery in MRA 3D reconstruction

A 45-year-old man was admitted in the hospital as he complained of dizziness and blurred vision on November 8, 2017. The patient was diagnosed with glioma in right frontal lobe and received tumorectomy 19 years ago. After surgery, he underwent whole brain irradiation plus focal irradiation over 14 times in half a month. He had a history of stroke 13 years ago presenting with left limb weakness. The exact mechanism of stroke was not confirmed. Assessment of intracranial arteries was not performed and antiplatelet drugs were not prescribed. The patient had no history of hypertension, diabetes mellitus, dyslipidemia, coronary artery disease, atrial fibrillation, smoking and alcohol abuse. Family history of stroke was not identified. The patient did not take any secondary prevention measures for cerebrovascular disease. At admission, neurological clinical examinations were normal except bilateral left hemianopia. The score of National Institutes of Health Stroke Scale (NIHSS) was 0. Magnetic resonance imaging (MRI) showed acute infarcts in temporal lobe and occipital lobe (Fig. 1).

**Fig. 2:**
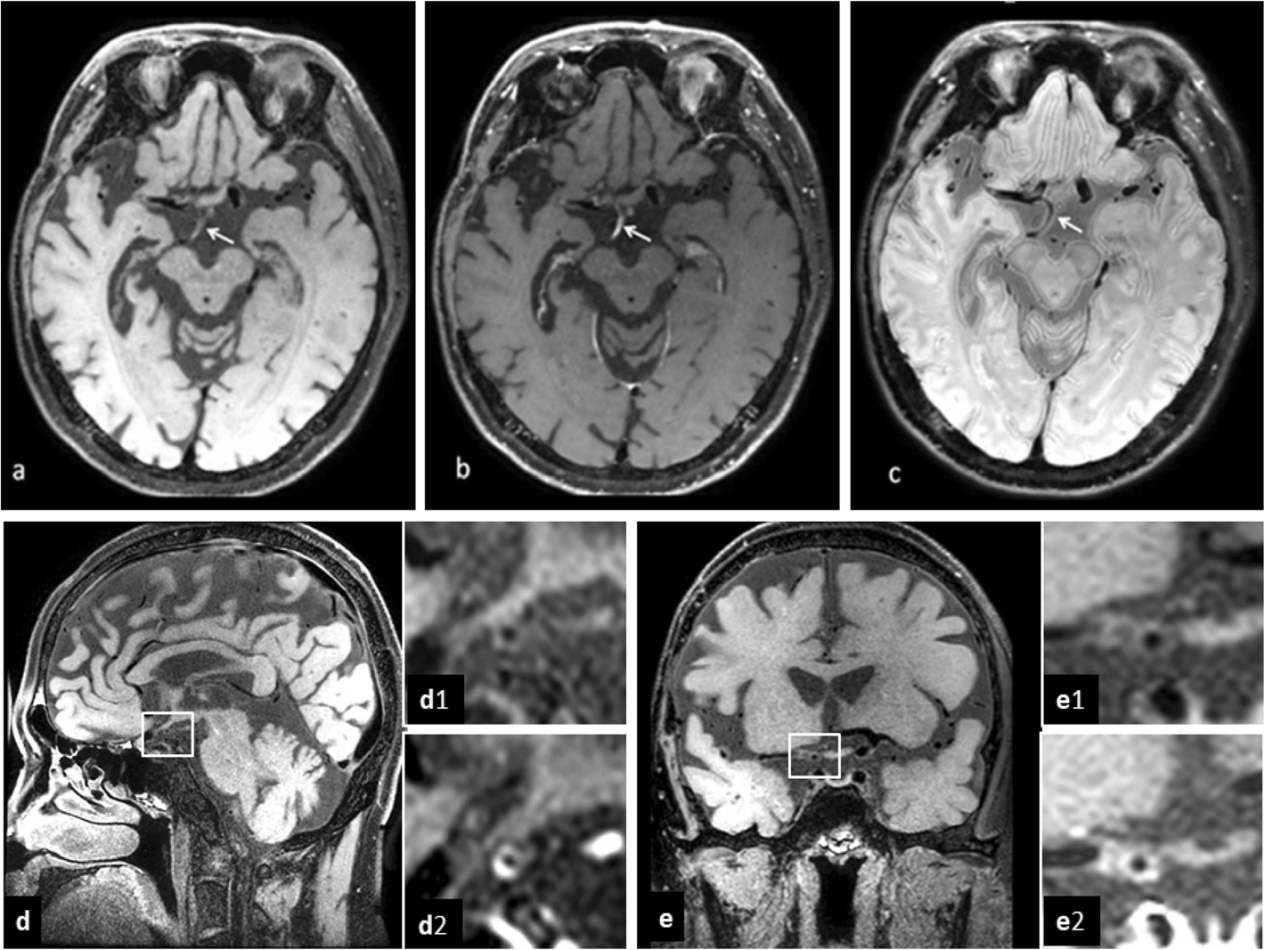
Brain high-resolution magnetic resonance imaging (HR-MRI). **a**-**c**: Mild T1 hyperintensity within the right posterior communicating artery wall in T1 pre-contrast HR-MRI, incomplete enhancement in T1 post-contrast HR-MRI and narrow lumen in PD-weighted HR-MRI. **d**-**e**: The right posterior communicating artery(**d**) and the right middle cerebral artery(**e**), concentric thickening of vascular wall in HR-MRI T1 sequence(d1, e1) and strong and concentric wall enhancement in T1 post-contrast HR-MRI(d2, e2)

In order to identify the cause of stroke, blood tests including blood routine examination, glucose, serum lipid, liver and renal function, coagulation, syphilis, HIV, auto-immune screen and thrombophilia were performed and results were normal. Electrocardiography and echocardiography were negative and cardiogenic cerebral embolism was excluded. In cerebrospinal fluid (CSF) examination, level of albumin and IgG were elevated (ALB = 740 mg/l, IgG = 69 mg/l). By MRI, multiple cerebral microbleeds (CMBs), lacunar infarcts in the brainstem and acute infarcts in temporal lobe and occipital lobe were found (Fig. 1). With the use of magnetic resonance angiography (MRA), we found that bilateral posterior cerebral artery originated from the bilateral internal carotid artery. And multiple localized stenosis in right middle cerebral artery and left posterior cerebral artery. We believed that right posterior cerebral artery was the responsible vessel of acute stroke. HR-MRI examination showed vascular inflammatory changes in multiple cerebral arteries, especially in the right middle cerebral artery and right posterior communicating artery. HR-MRI showed vessel wall thickening in T1-weighted imaging, narrow lumen in proton density imaging and vessel wall concentric enhancement in contrast-enhanced T1- weighted imaging (Fig. 2). For the multiple CMBs and vascular inflammatory changes, prednisone (30 mg/day) was prescripted to our patient. Followed by telephone, clinical symptoms disappeared after 3 months.

## Discussion and conclusions

We report a young stroke patient with history of radiotherapy. We considered right posterior cerebral artery as the responsible vessel. HR-MRI showed vascular inflammatory changes in vessel wall, without dissection and premature atherosclerosis. As there was no history of infectious, auto-immune and inherited disease in our patient, we believed that intracranial artery stenosis was caused by radiation-induced arteriopathy.

Multiple CMBs were detected in our patient. CMB is a common type of radiation-induced vascular complication, indicating microvascular injury. Previous study showed that CMB could progress with each year from time of radiotherapy, which predominately located in bilateral occipital lobes. In our patient, we believed that CMB is related with radiotherapy but not cerebral amyloid angiopathy (CAA) because of patient’s young age and absence of cerebral hemorrhage and cognitive impairment.

Radioactive damage to brain tissues is a long-term process. However, vascular changes after radiotherapy over 15 years were rarely reported. In our patient, vascular inflammatory changes were still detected by HR-MRI after his first radiotherapy 19 years ago. The exact mechanisms were not fully understood. An autoimmune inflammatory changes induced by radiation has been proposed [7]. Vascular changes has been considered as key feature of delayed radiation injury, which includes marked atypia or loss of endothelial cells, vascular fibrosis leading to luminal occlusion, and fibrinoid vascular necrosis. Vascular changes after radiotherapy may be a dynamic process involving increased expression of vascular endothelial growth factor (VEGF), blood brain barrier damage, oxidative stress and inflammation. Therefore, radiation-induced arteriopathy may be an important cause of stroke in patients with radiotherapy and more attention should be paid to it.

HR-MRI has been considered as a useful tool for distinguishing intracranial vascular lesions by displaying both lumen and vessel wall [8]. Many etiologies can lead to similar lumen stenosis, including intracranial atherosclerosis, vasculitis, intracranial arterial dissection and moyamoya disease. On HR-MRI, eccentric and irregular plaques are the characteristic finding of atherosclerosis, whereas patterns of concentric arterial wall thickening and homogeneous enhancement of the vessel wall indicate vasculitis. The decreasing vessel wall enhancement after anti-inflammatory treatment also supports the diagnosis of vasculitis [9, 10]. With HR-MRI, intimal flap and double lumen are typical imaging findings in patients with intracranial artery dissections, and intramural hematoma and aneurysmal dilatation can also be detected [11, 12]. A decrease in the outer diameter and concentric and weaker enhancement in the bilateral terminus of the internal cerebral artery, proximal anterior cerebral artery, or middle cerebral artery are characteristics of moyamoya disease [13]. The right middle cerebral artery and posterior communicating artery of our patient, concentric thickening of vascular wall in HR-MRI T1 sequence, strong and concentric wall enhancement in T1 post-contrast HR-MRI, the whole features of multiple cerebral arteries support the diagnosis of central nervous system vasculitis (CNSV). And secondary CNSV was considered in our case because of negative blood tests and history of radiotherapy.

Effective treatments of radiation-induced brain injury are limited. In our case, the patient received low-dose hormone therapy for 3 months in order to regulate autoimmune inflammation. The clinical symptoms disappeared after 3 months. In the latest report, addition of an anti-angiogenic agent enzastaurin decelerated appearance of CMBs, showing radioprotective effect on microvasculature [14]. However, the exact mechanism is still largely unknown and more studies are needed.

In conclusion, radiation-induced cerebrovascular damage is a long-lasting progress. HR-MRI can provide sensitive and accurate diagnostic assessment of radiation-induced arteritis and can be used as a useful tool for the screening of causes of cryptogenic stroke.

## Data Availability

All data have been presented within the manuscript and additional supporting files.

## Abbreviations

ADC: Apparent diffusion coefficient
CAA: Cerebral amyloid angiopathy
CMBs: Cerebral microbleeds
CNSV: Central nervous system vasculitis
CSF: Cerebrospinal fluid
DWI: Diffusion weighted imaging
FLAIR: Fluid attenuated inversion recovery
HIV: Human immunodeficiency virus
HR-MRI: High-resolution magnetic resonance imaging
MRA: Magnetic resonance angiography
MRI: Magnetic resonance imaging
NIHSS: National Institutes of Health Stroke Scale
SWI: Susceptibility weighted imaging
VEGF: Vascular endothelial growth factor
